# Calculating Variations in Biological Effectiveness for a 62 MeV Proton Beam

**DOI:** 10.3389/fonc.2016.00076

**Published:** 2016-04-06

**Authors:** Mario Pietro Carante, Francesca Ballarini

**Affiliations:** ^1^Physics Department, University of Pavia, Pavia, Italy; ^2^Istituto Nazionale di Fisica Nucleare – Sezione di Pavia, Pavia, Italy

**Keywords:** cell death, chromosome aberrations, protons, hadron therapy, biophysical models, Monte Carlo simulations, relative biological effectiveness

## Abstract

A biophysical model of radiation-induced cell death and chromosome aberrations [called BIophysical ANalysis of Cell death and chromosome Aberrations (BIANCA)] was further developed and applied to therapeutic protons. The model assumes a pivotal role of DNA cluster damage, which can lead to clonogenic cell death following three main steps: (i) a DNA “cluster lesion” (CL) produces two independent chromosome fragments; (ii) fragment mis-rejoining within a threshold distance *d* gives rise to chromosome aberrations; (iii) certain aberration types (dicentrics, rings, and large deletions) lead to clonogenic inactivation. The yield of CLs and the probability, *f*, that a chromosome fragment remains un-rejoined even if other fragment(s) are present within *d*, were adjustable parameters. The model, implemented as a MC code providing simulated dose–responses directly comparable with experimental data, was applied to pristine and modulated Bragg peaks of the proton beam used to treat eye melanoma at INFN-LNS in Catania, Italy. Experimental survival curves for AG01522 cells exposed to the Catania beam were reproduced, supporting the model assumptions. Furthermore, cell death and chromosome aberrations at different depths along a spread-out Bragg peak (SOBP) dose profile were predicted. Both endpoints showed an increase along the plateau, and high levels of damage were found also beyond the distal dose fall-off, due to low-energy protons. Cell death and chromosome aberrations were also predicted for V79 cells, in the same irradiation scenario as that used for AG01522 cells. In line with other studies, this work indicated that assuming a constant relative biological effectiveness (RBE) along a proton SOBP may be sub-optimal. Furthermore, it provided qualitative and quantitative evaluations of the dependence of the beam effectiveness on the considered endpoint and dose. More generally, this work represents an example of therapeutic beam characterization avoiding the use of experimental RBE values, which can be source of uncertainties.

## Introduction

According to the Particle Therapy Co-operative Group^1^, 49 proton therapy centers were operating and 32 were under construction in June 2015. The rationale of using protons instead of conventional radiotherapy relies on the ability of these particles to reduce the dose to normal tissues, thanks to the dose localization in the (spread-out) Bragg peak (SOBP) (1). In addition to different types of tumors, protons can also be used to treat non-cancer diseases, such as arteriovenous malformations (2).

Protons are usually considered low-LET radiation, and a constant relative biological effectiveness (RBE) of 1.1, mainly derived from animal experiments, is generally applied in the clinical practice. However, both *in vitro* and *in vivo* studies indicate that proton effectiveness increases with decreasing energy, which is increasing LET. This implies an increase of effectiveness with depth along the SOBP, as well as an extension of the biologically effective range. *In vivo*, the average RBE at mid-SOBP is ~1.1, ranging from 0.7 to 1.6 (3); *in vitro* data on clonogenic cell survival indicate an average value at mid-SOBP of ~1.2, ranging from 0.9 to 2.1 (3). Furthermore, the RBE depends not only on the particle energy but also on many other factors, including dose, dose-rate, cell type, and biological endpoint. For instance, both *in vitro* and *in vivo* data show a significant RBE increase for lower fractional doses [e.g., Ref. (4, 5)], especially for cells and tissues with low α/β ratio (6). This may be one of the reasons why *in vivo* experiments, most of which have been performed at higher doses, suggest lower RBE values with respect to *in vitro* studies. It should also be considered that, although the main endpoint of interest for tumor cells is cell death, other endpoints (e.g., mutations, non-lethal chromosome aberrations, etc.) might be relevant for normal tissues.

Although clinical results do not indicate that the use of a constant RBE is incorrect, no trials specifically targeted RBE variations; moreover, tighter treatment margins may increase the importance of taking into account such variations (7). Applying a constant RBE of 1.1 may lead to an underestimation of the damage to normal tissues, especially for treatments involving organs at risk just beyond the tumor, such as the retina for eye tumors and the heart for (left) breast tumors, which are becoming a major application of protontherapy [e.g., Ref. (8)]. On the other side, the currently available RBE data might be insufficient to support a change in clinical practice (7). Incorporating variations in biological effectiveness without directly considering the RBE may be an alternative strategy. For instance, it has been suggested that LET distributions in the patient can be used to guide treatment plan optimization (9).

In this framework, a biophysical model of radiation-induced cell death and chromosome aberrations called BIophysical ANalysis of Cell death and chromosome Aberrations (BIANCA) (10–13) was developed at the University of Pavia and INFN-Pavia, Italy. The model, which in the last few years has been tested against *in vitro* cell survival data and has been applied in the framework of Boron Neutron Capture Therapy (14), assumes that DNA cluster damage can lead to chromosome aberrations and that some aberration types lead to clonogenic cell death. This approach allows calculating cell survival without relying on the concept of RBE. Furthermore, the capability of the model to calculate the induction of different chromosome aberration types, in addition to cell death, makes it suitable for applications in the framework of normal tissue damage evaluation, since some chromosome aberrations are known to be related to the risk to normal tissues (15). In the present work, after comparing simulated dose–response curves for chromosome aberrations with experimental data taken from the literature, the model was applied to the 62-MeV proton beam used to treat ocular melanoma at the CATANA facility of INFN-LNS in Catania, Italy (16). Experimental survival curves taken from the literature (17) for AG01522 cells exposed to pristine and SOBPs from the CATANA beam were reproduced, and cell death and chromosome aberrations were calculated for different depth positions along a SOBP. Finally, cell death and chromosome aberrations were predicted for another cell line (V79) exposed to the same dose profile used for AG01522 cells.

## Materials and Methods

### Model Assumptions

The BIANCA model is based on the following assumptions: (1) radiation induces DNA “cluster lesions” (CLs), and each CL gives rise to two independent chromosome fragments; (2) two chromosome fragments can undergo rejoining only if their *initial* distance is smaller than a threshold distance *d*, leading to chromosome aberrations in case of mis-rejoining (accidental un-rejoining is allowed); and (3) dicentrics, rings, and large deletions lead to clonogenic cell death.

A characterization of the “critical” DNA damage(s), which is damage type(s) that can lead to important endpoints such as chromosome aberrations and cell death, is still an open question in radiobiology. Therefore, we chose not to provide a definition for the quantity “CL,” leaving the mean number of CLs per unit radiation dose and per unit DNA mass (that is, the mean number of CLs per Gy and per Dalton, which can be easily converted into CLs per Gy and per cell) as an adjustable parameter. In a previous work (13), CL yields for different radiation qualities showed good agreement with yields of kilo-base-pair (kbp) DNA fragments, suggesting that DSB clusters at the kbp scale, possibly in addition to other levels of clustering, may play a relevant role.

Assumption (2) reflects the fact that fragment rejoining is thought to be distance dependent. The adoption of a step function, rather than a continuously decreasing function, implicitly takes into account the existence in the cell nucleus of repair centers, where DSBs should migrate for repair. For instance, 1–2 μm DSB migration distances have been estimated for MCF10A epithelial cells (18). In previous works [e.g., Ref. (13)], where the threshold distance *d* was considered as an adjustable parameter, a *d* value of 5 μm led to good agreement with experimental survival curves for AG1522 human fibroblasts and V79 hamster fibroblasts exposed to different radiation qualities. However, this value seems to be larger than most estimations available in the literature. In the present work, a different approach was adopted, setting the value of *d* equal to the mean distance between two adjacent chromosome territories (which resulted to be 3.0 μm for AG cells and 3.6 μm for V79 cells), basing on the idea that repair mainly takes place in small channels separating adjacent chromosome domains (19). The expression “chromosome territories” refers to distinct regions of the cell nucleus, with negligible reciprocal overlapping, where the various chromosomes are localized during interphase, that is most of the cell cycle. According to this approach, *d* is no more an adjustable parameter, but is fixed *a priori* basing on the specific features of the considered cell nucleus (i.e., nucleus shape and dimensions and number of chromosomes).

In previous works, a chromosome fragment having at least one potential partner for rejoining (that is, at least another fragment within the threshold distance *d*) was assumed to undergo rejoining with 100% probability. On the contrary in the present work, we considered a more realistic scenario where a fragment, even if one or more potential “partners” are available within *d*, has a given probability *f* of remaining un-rejoined. This assumption is consistent with studies indicating that a certain fraction of exchange-type chromosome aberrations are “incomplete,” i.e., not all the involved chromosome fragments are finally rejoined [e.g., Ref. (20–22)]. The observed probability of unrejoining tends to be cell-line-dependent, since in general radiosensitive cells show higher frequencies of deletions with respect to normal or radioresistant cells. For instance, in ataxia-telangiectasia (A-T) cells exposed to X-rays, the fraction of un-rejoined breaks was five to six times higher than that for normal fibroblasts (23). Concerning a possible dependence on radiation quality, contradicting results can be found in the literature. While some works report an increase of incomplete exchanges with LET [e.g., Ref. (21)], others do not indicate a LET-dependence [e.g., Ref. (20, 22)]. For the sake of simplicity, in the present work we assumed that, for a given cell line, the value of *f* was cell line dependent but LET independent.

Assumption (3) derives from the relationship between chromosome aberrations and cell death shown by many works available in the literature. In particular, for AG1522 fibroblasts exposed to X-rays, Cornforth and Bedford (24) found a one-to-one relationship between the mean number per cell of “Lethal Aberrations” (defined as dicentrics plus rings plus deletions visible in Giemsa) and –lnS, where S is the fraction of surviving cells. According to another work, an analogous relationship may hold for V79 cells as well (25).

### Dose–Response Simulations

Like in previous works, AG1522 cell nuclei were modeled as cylinders with elliptical base (height: 4 μm; major axis: 20 μm; minor axis: 10 μm), and V79 cell nuclei were modeled as cylinders with circular base (height and radius: 6 μm). A discussion on these choices can be found in Carante et al. (13). Each interphase chromosome territory was represented as the union of adjacent cubic voxels of 0.2 μm side to obtain chromosome territories with volume proportional to their DNA content. The various territories were simultaneously constructed step-by-step, with the first step consisting of random selection of a “starting voxel” for each chromosome territory. In each of the subsequent steps, a new voxel was assigned to each territory; the new voxel was randomly selected among the six closest neighbors of the voxel that was assigned to that territory in the preceding step. After constructing the various chromosome territories, each of the voxels assigned to a given territory was associated with one of the two chromosome arms, applying a probability proportional to the arm DNA content.

To simulate the exposure to a given dose of X-rays, a CL yield (mean number of CLs per Gy and per cell) was multiplied by that dose to obtain the mean number of CLs per cell. For each cell, which is for each run of the code, an “actual” number of CLs was then extracted from a Poisson distribution, and those CLs were distributed within the nucleus volume uniformly, since X-rays are sparsely ionizing radiation. For protons, the simulation for a given dose started calculating the mean number of (primary) particles traversing the cell nucleus, with direction parallel to the axis of the cylinder representing the nucleus. Such mean number was calculated by *n* = *S *×* D/(0.16 *×* L)*, where *S* is the nucleus cross-sectional area in square micrometer, *D* is the absorbed dose in Gy, *L* is the radiation LET in keV/μm, and 0.16 is a factor coming from the conversion between eVs and Joules. For each cell, an “actual” number of nucleus traversals was then extracted from a Poisson distribution having mean value *n*, and for each traversal an entrance point in the nucleus was randomly selected. The mean number of CLs per nucleus traversal was then calculated multiplying the nucleus traversal length (in micrometer) by the mean number of CLs per micrometer. The latter was obtained by *CL*/μm = 0.16 × (*CL*⋅**Gy^−1^⋅cell^−1^) × *L*/*V*, where *V* is the cell nucleus volume in cubic micrometer, *L* is the radiation LET in keV/μm, and 0.16 is the conversion factor mentioned above. For each nucleus traversal, an “actual” number of CLs was extracted from a Poisson distribution, and these CLs were uniformly distributed along the segment representing that traversal.

The subsequent simulation steps consisted of the following: identification of the chromosome and the chromosome-arm that was hit by each CL; rejoining of chromosome fragments within the threshold distance *d*; scoring of lethal aberrations (dicentrics, rings and deletions visible in Giemsa); and calculation of the corresponding surviving fraction. Chromosome fragments having a DNA content smaller than 3 Mega-base-pairs (Mbp) were assumed as not visible in metaphase, as reported by Cornforth and Bedford (24). A discussion on the role of this value can be found in Carante et al. (13). For each dose, the code was run 10,000 times, allowing to obtain a relative error smaller than 2%. The repetition for different doses provided simulated dose–response curves for chromosome aberrations or cell survival, directly comparable with experimental data.

## Results and Discussion

### Chromosome Aberrations

According to the approach adopted in the current version of the model, (clonogenic) cell death depends on chromosome aberrations, in particular the so-called “lethal aberrations” (dicentrics, rings, and deletions). Comparisons between calculated and experimental yields of different chromosome aberration types have been published in previous works [e.g., Ref. (12, 26–29)]. However, new comparisons were performed following the introduction of some modifications: in the present work, as mentioned above, the threshold distance for chromosome fragment rejoining, *d*, was fixed as the mean distance between two adjacent chromosome territories, and a chromosome fragment was allowed to remain un-rejoined (with probability *f*) also when possible “partners” were present within *d*.

**Figure 1:**
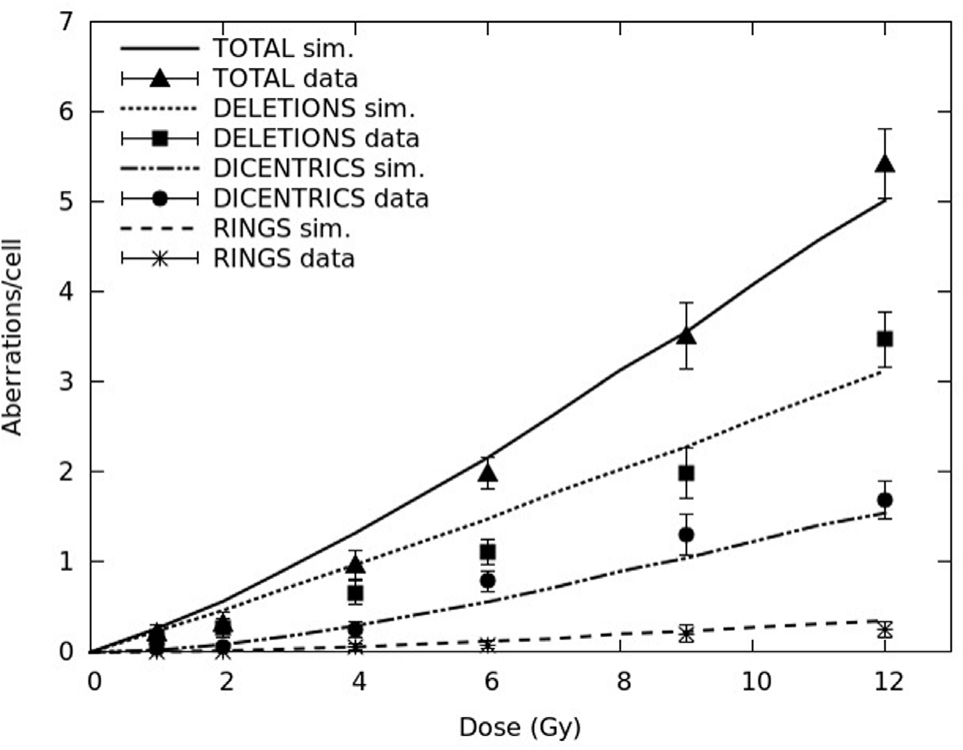
**Yields (mean number per cell) of different aberration types (dicentrics, rings, and deletions, as well as total lethal aberrations) in AG1522 primary normal human fibroblasts exposed to different doses of X-rays**. The lines are simulation outcomes, the points are experimental data taken from Cornforth and Bedford (24).

A detailed and systematic discussion on this issue is beyond the scope of the present work, and will be object of a separate paper. As an example, Figure 1 shows dose–response curves for dicentrics, rings, and deletions (both separately and summed up to give total aberrations) induced in AG1522 cells exposed to X-rays. The lines are simulation outcomes, the points are experimental data taken from the literature (24). The error bars associated with the experimental points, which represent 95% confidence about means as reported in Ref. (24), were calculated from the aberration yields and the number of analyzed cells reported in table 2 of the experimental paper. Both for dicentrics and rings, the calculated aberration yields were within the experimental errors, with the only exception of dicentrics at 6 Gy. Incidentally, the capability of reproducing separately the yields of dicentrics and rings supports the assumption adopted for *d*, since higher *d* values overestimated the ratio of dicentrics to rings (the so-called “*F*-ratio”), whereas lower *d* values underestimated the *F*-ratio (results not shown). Concerning deletions, the question seems more qualitative than quantitative: while the simulated response is basically linear, the experimental response shows a non-negligible quadratic component. This can be explained considering that in the simulations most deletions were “terminal deletions,” which being due to a single chromosome break involve a single-particle mechanism proportional to dose, whereas most experimental deletions were of the “interstitial” type, which requiring two chromosome breaks (also) involves a two-particle mechanism proportional to the square of dose. The observation of so many interstitial deletions following exposure to X-rays, which are sparsely ionizing radiation, is not easy to explain. One possible reason might be related to the particular experimental protocol, according to which the cells were sub-cultured for 24 h after irradiation.

The curves reported in Figure 1 were obtained with a *f* value of ~0.2, and a CL yield of ~1.3 CL Gy^−1^⋅cell^−1^. This value is lower than the value used to reproduce total aberration yields in previous works [e.g., Ref. (12)] where parameter *f* was not introduced, which means *f* = 0. This can be explained considering that in the present work, where a chromosome fragment is allowed to remain un-rejoined also in presence of potential partners within the threshold distance, the yield of deletions – and thus of total aberrations – increased, implying that a lower CL yield was sufficient to get the same yield of total aberrations. Although it was possible to reproduce the yields of total aberrations also assuming that *f* = 0 (12), at most doses the yields of deletions were underestimated by a factor ~2, and the yields of dicentrics were overestimated, again by a factor ~2. On the contrary, as shown in Figure 1, the introduction of a *f* value higher than 0 allowed obtaining a good agreement not only with total aberrations as a whole (upper curve) but also with dicentrics, rings, and deletions considered separately (three lower curves). A determination of the “best” *f* value was beyond the scope of the present work. However, it is worth reporting that attempting to reproduce the (experimental) yields of total aberrations with lower *f* values (and higher CL yields) led to an underestimation of deletions associated with an overestimation of dicentrics, whereas higher *f* values (with lower CL yields) led to an overestimation of deletions associated with an underestimation of dicentrics (results not shown).

### Survival Curves

The model was then applied to cell survival, focusing on protons due to their wide use in hadron therapy. The first step of the work consisted of reproducing experimental survival curves obtained with the 62-MeV proton beam available at the CATANA ocular melanoma facility of INFN-LNS in Catania, Italy (16, 17). In that experiment (17), AG01522 primary normal human fibroblasts were exposed to six pristine Bragg peaks (with minimum and maximum water-equivalent depth of 1.7 and 30.7 mm, respectively) and at six depth positions along a SOBP (with minimum and maximum water-equivalent depth of 1.5 and 31.2 mm, respectively). After irradiation, the cells were immediately trypsinized, counted, seeded, and incubated to allow for macroscopic colony formation; colonies consisting of at least 50 cells were scored as viable. Further details can be found in the original paper (17).

**Figure 2:**
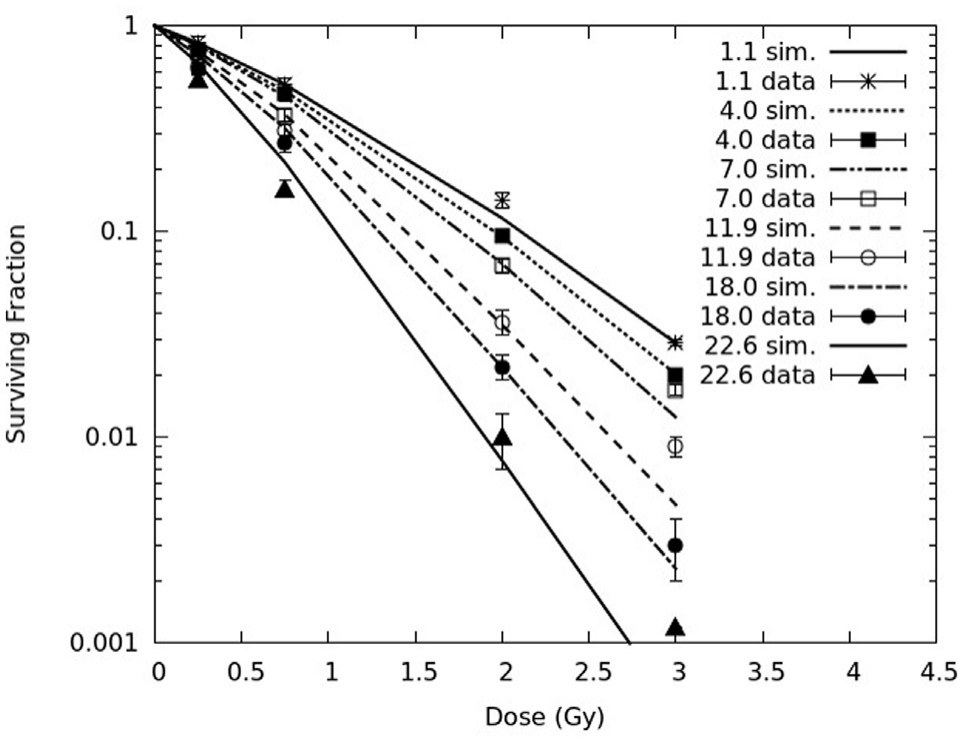
**Cell survival curves for AG01522 primary normal human fibroblasts exposed to six pristine proton Bragg peaks (corresponding to the following LET values: 1.1, 4.0, 7.0, 11.9, 18.0, and 22.6 keV/μm)**. The lines are simulation outcomes, the points are experimental data taken from Chaudhary et al. (17).

Figure 2 reports simulated survival curves for the six pristine peaks (corresponding to the following LET values: 1.1, 4.0, 7.0, 11.9, 18.0, and 22.6 keV/μm), together with the experimental data for comparison and their error bars, which represent one SD. Raw numbers were obtained from the authors of the experimental work. All simulations were performed adopting the same value of *f* used to calculate chromosome aberrations in AG human fibroblasts, which is 0.2. On the contrary, the yield of CLs, which depends on radiation quality, was adjusted separately for each curve. The curves reported in Figure 2 were obtained using CL yields in the range ~4.1–8.0 CL Gy^−1^⋅cell^−1^, increasing with the radiation LET. The increase with LET is consistent with the clustering nature of CLs [e.g., Ref. (28, 30)]. Analogous to chromosome aberrations, the CL yields used to obtain the curves shown in Figure 2 were (slightly) lower with respect to those used in previous works. Again, this is related to the introduction of parameter *f*, which implying higher yields of lethal aberrations, also implies lower survival levels; as a consequence, a lower CL yield was sufficient to get the same survival curve for a given radiation quality. In general, the simulation outcomes showed satisfactory agreement with the experimental data. In some cases, typically for curves corresponding to higher LET values, there was a tendency to underestimate the experimental survival at the highest considered dose, which was 3 Gy. This issue is under investigation. More specifically, for 1.1 and 4.0 keV/μm the value of the reduced chi-square was around 1. Higher values were found for the other four curves, mainly due to the point at 3 Gy; however, at least in two cases (7.0 and 22.6 keV/μm), the simulations were close to the fit performed by the authors, since the relative difference between calculated and fitted survival was smaller than 20%.

**Figure 3:**
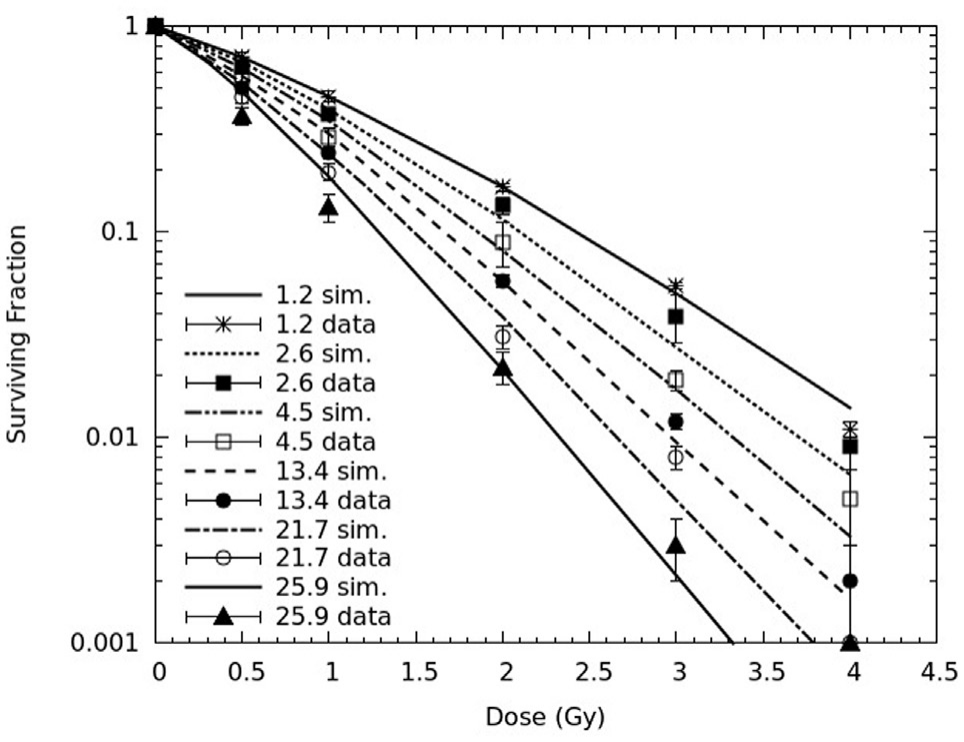
**Cell survival curves for AG01522 primary normal human fibroblasts irradiated at six depth positions along a proton SOBP (corresponding to the following dose-averaged LET values: 1.2, 2.6, 4.5, 13.4, 21.7, and 25.9 keV/μm)**. The lines are simulation outcomes, the points are experimental data taken from Chaudhary et al. (17).

**Figure 4:**
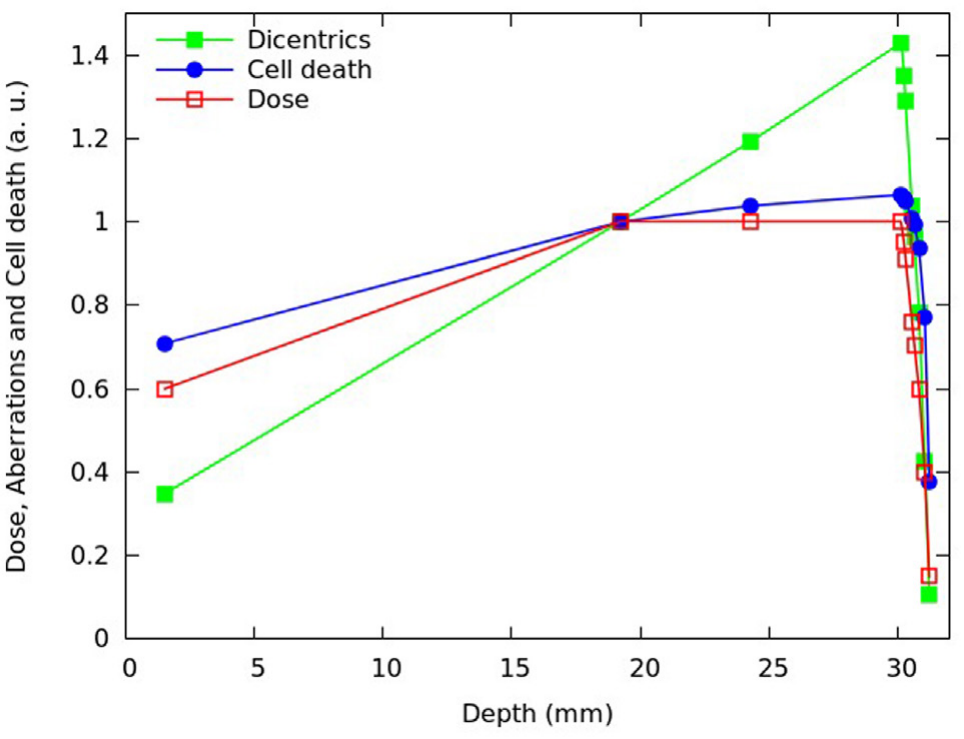
**Calculated fraction of inactivated cells (blue symbols) and calculated mean number of dicentrics per cell (green symbols) at different depths of the SOBP dose profile reported in Chaudhary et al. (17), which is also shown in the figure (red symbols)**. Each quantity was normalized with respect to the proximal position. The lines are simply guides for the eye.

**Figure 5:**
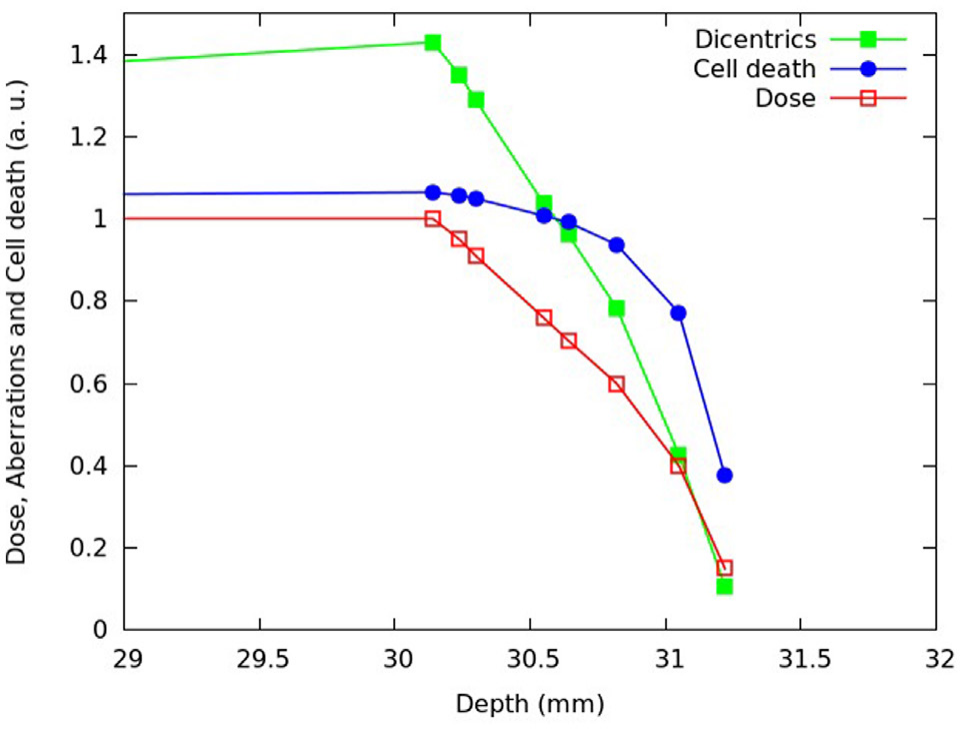
**Distal and fall-off region of the SOBP shown in Figure 4**.

**Figure 6:**
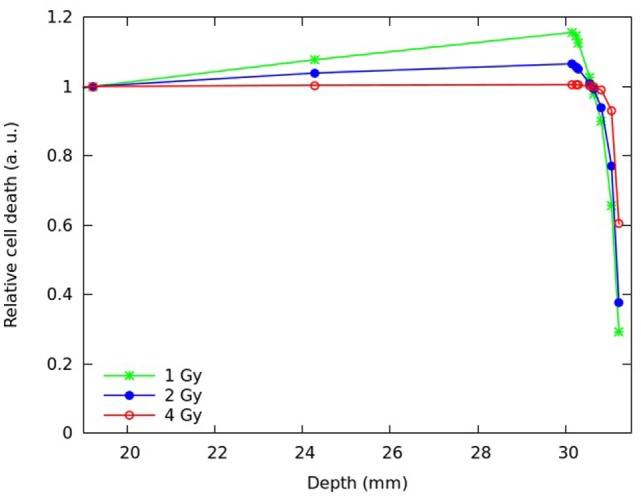
**Predicted fraction of inactivated cells at different depths along the SOBP, assuming a plateau dose of 1 Gy (green symbols), 2 Gy (blue symbols), or 4 Gy (red symbols)**. Each quantity was normalized with respect to the proximal position. The lines are simply guides for the eye.

**Figure 7:**
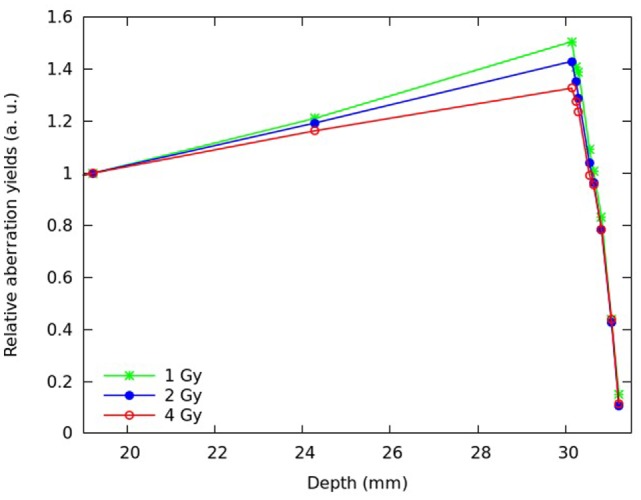
**Predicted mean number of dicentrics per cell at different depths along the SOBP, assuming a plateau dose of 1 Gy (green symbols), 2 Gy (blue symbols), or 4 Gy (red symbols)**. Each quantity was normalized with respect to the proximal position; the lines are simply guides for the eye.

**Figure 8:**
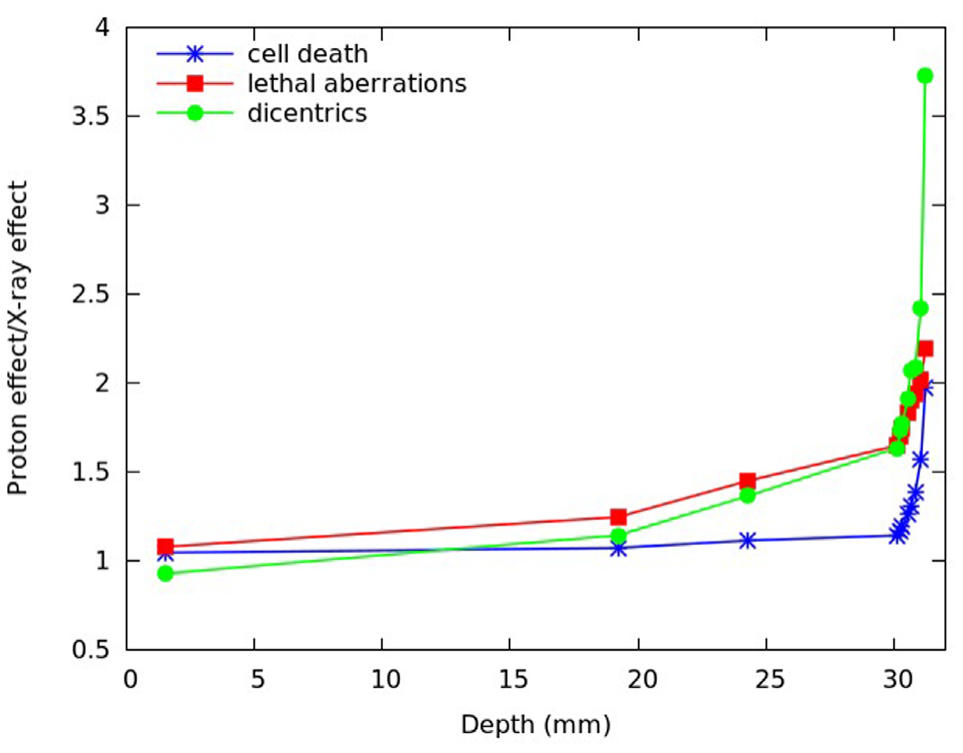
**R_I_ (ratio between proton-induced and X-ray-induced cell inactivation after the same dose, blue symbols), R_LA_ (ratio between proton-induced and X-ray-induced lethal aberrations after the same dose, red symbols), and R_DIC_ (ratio between proton-induced and X-ray-induced dicentrics after the same dose, green symbols) calculated at different depths of the SOBP dose profile used in Chaudhary et al. (17), assuming a plateau dose of 2 Gy**. The lines are simply guides for the eye.

**Figure 9:**
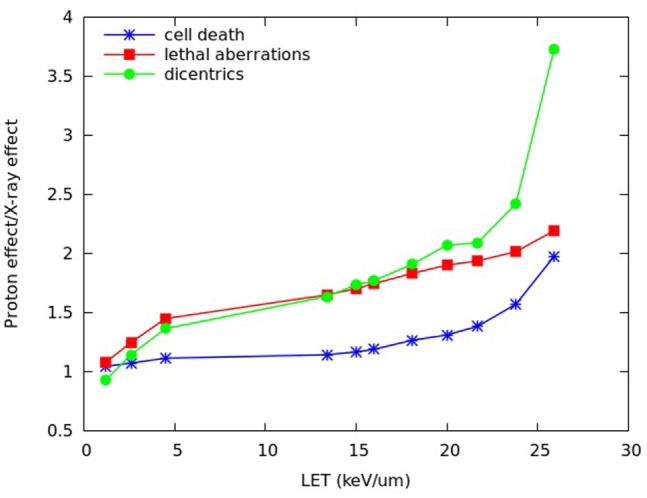
**R_I_ (ratio between proton-induced and X-ray-induced cell inactivation after the same dose, blue symbols), R_LA_ (ratio between proton-induced and X-ray-induced lethal aberrations after the same dose, red symbols), and R_DIC_ (ratio between proton-induced and X-ray-induced dicentrics after the same dose, green symbols) for the (dose-averaged) LET values corresponding to the depth positions considered in Figure 8**. The lines are simply guides for the eye.

In Figure 3, simulated survival curves are compared with the experimental data obtained by Chaudhary et al. at the six depth positions along the SOBP, corresponding to the following dose-averaged LET values: 1.2, 2.6, 4.5, 13.4, 21.7, and 25.9 keV/μm. Again, all simulations were performed without changing the value of *f*, whereas the CL yield was adjusted separately for each radiation quality, that is for each curve. Despite a tendency to underestimate the experimental survival at high doses, already mentioned for the pristine peaks, CL yields in the range ~3.7–6.4 CL Gy^−1^⋅cell^−1^, increasing with LET, led to a satisfactory agreement with the experimental curves. Analogous to the results for the pristine peaks, also for the spread-out peak the agreement between simulations and experiments was particularly good for the lower LET curves, since a reduced chi-square around 1 was obtained for 1.2, 2.6, and 4.5 keV/μm. Higher (reduced) chi-square values were found for 13.4, 21.7, and 25.9 keV/μm, mainly due to the points at the highest doses (3 and 4 Gy). However, with the only exception of the point at 3 Gy for the 21.7 keV/μm curve, the relative difference between calculated and fitted survival was not larger than 20%. It is also worth mentioning that, since the higher LET values refer to the descending part of the SOBP, where the doses are lower, the underestimation of the experimental survival at high doses of higher LET did not lead to important consequences on the predictions of cell killing and chromosome aberrations along the SOBP dose profile that will be shown in Figures 4–9.

Interestingly, the CL yields used for the curves reported in Figure 3 were lower than the CL yields used for the pristine peaks (Figure 2). This is consistent with the higher RBE observed in the experimental work for the pristine peaks with respect to the SOBP (17), and may be related to the fact that a SOBP consists of a mixed radiation field that can only be associated with an average LET, rather than a single LET value. This issue has been discussed for carbon beams by Belli et al. (31), who suggested that these differences between SOBP and monoenergetic beams may also depend on the specific cell line, in addition to the ion type. More specifically, according to these authors, a systematic deviation may be related to the averaging procedures in the presence of a LET distribution along the SOBP. Moreover, if this distribution is large enough to include high-LET values falling close to or beyond the RBE maximum, the so-called “overkilling effect” might result in a further decrease in biological effectiveness (31).

### Applications for Protontherapy

After reproducing the survival curves reported in Chaudhary et al. (17) for the pristine peaks and the various SOBP positions, the model was applied to investigate the depth- and dose dependence of the beam effectiveness along the SOBP, in terms of both cell death and chromosome aberrations. For different depths in water of the SOBP dose profile reported in Chaudhary et al. (17), Figures 4 and 5 report the relative fraction of inactivated cells and the relative yield of dicentrics, assuming a dose of 2 Gy in the plateau region. The term “relative” means that each quantity was normalized with respect to the proximal point. For the six depth positions considered in the experimental work (i.e., 1.52, 19.22, 24.28, 30.14, 30.82, and 31.22 mm), the cell killing calculations did not add substantial information with respect to the experimental work. However, the model allowed predicting the fraction of surviving cells also for other positions, with focus on the dose fall-off region that can be critical for normal tissue damage (see Figure 5). Moreover, the model provided predictions of chromosome aberrations, which were not investigated in Chaudhary et al. (17). This information may be useful in the framework of normal tissue damage evaluation, since certain types of chromosome aberrations (typically, reciprocal translocations) are known to be related to cell conversion to malignancy (15). For this reason, dicentric yields were shown in Figures 4 and 5: dicentric yields are thought to be not significantly different than the yields of reciprocal translocations, which are the symmetrical counterpart of dicentrics among inter-chromosomal simple exchanges.

Consistent with the experimental data reported in Chaudhary et al. (17) and with other works available in the literature [e.g., Ref. (4, 32, 33)], the beam effectiveness – both for cell death and for chromosome aberrations – was found to increase with depth along the plateau, and high levels of biological damage were also found beyond the distal fall-off. For instance at ~31 mm in water, where the physical dose was about 40% of the proximal dose, the fraction of inactivated cells was almost 80% of the fraction of inactivated cell at the proximal position. This can be explained taking into account that, as protons slow down, their LET increases leading to a higher biological effectiveness. Furthermore, the (relative) increase in chromosome aberrations with increasing depth along the plateau was more pronounced with respect to cell killing: while cell killing increased by a factor ~1.1, the yield of dicentrics (and, thus, reciprocal translocations) in the distal position was more than 1.4 times higher with respect to the proximal position. This is an example of dependence of biological effectiveness on the considered endpoint.

Predictions of cell death and chromosome aberrations were also performed assuming different plateau doses. Figures 6 and 7 report predictions for the fraction of inactivated cells (Figure 6) and the mean number of dicentrics per cell (Figure 7) at different depths of the SOBP, assuming a plateau dose of 1 or 4 Gy. For comparison, the figure also reports the results for 2 Gy. Again, the results were normalized with respect to the proximal position.

Increasing the physical dose (from 2 to 4 Gy) reduced the increase in biological effectiveness along the plateau, whereas decreasing the dose (from 2 to 1 Gy) led to an even more pronounced increase in effectiveness. This is consistent with the well-known dose-dependence of RBE, which tends to be higher at lower doses and vice versa. However, while for cell death, the highest considered dose (4 Gy) led to an almost flat biological effectiveness along the plateau; for chromosome aberrations, even that dose implied an increase in effectiveness.

To compare the effectiveness of protons with that of X-rays, the ratio between the level of effect (cell death or chromosome aberrations) induced by a given dose of protons and the level of effect induced by the same dose of X-rays was also investigated for different positions along the SOBP dose profile. Although this quantity has not the same meaning as the RBE, which is defined as the iso-effect ratio between the X-ray dose and the proton dose, both these ratios reflect variations in biological effectiveness. Figure 8 reports, for different depths along the SOBP dose profile assuming a plateau dose of 2 Gy, the calculated ratio between proton-induced cell death (i.e., fraction of inactivated cells) and cell death induced by the same dose of X-rays. This ratio will be called R_I_, where “I” means “inactivation.” The figure also reports the ratio between the yield of lethal aberrations (i.e., mean number of lethal aberrations per cell) induced by protons and the yield of lethal aberrations induced by the same dose of X-rays, which will be called R_LA_, as well as the ratio between the yield of dicentrics induced by protons and the yield of dicentrics induced by the same dose of X-rays, which will be called R_DIC_.

As expected, all these ratios increased with depth due to the increase in proton LET. However, their depth dependence showed different features. In particular, R_DIC_ (ratio between proton- and X-ray dicentrics) increased up to more than 3.5, whereas R_LA_ (ratio between proton- and X-ray lethal aberrations) and R_I_ (ratio between proton- and X-ray cell inactivation) increased up to about 2. Again, this is an example of different effectiveness when different endpoints – even different types of chromosome aberrations – are considered. The fact that dicentrics, considered as representative of reciprocal translocations, showed a more pronounced increase with respect to lethal aberrations and cell death may have implications in the evaluation of the risk to normal tissues.

In Figure 9, the same quantities reported in Figure 8, that is R_I_, R_LA_, and R_DIC_, are plotted as a function of the (dose-averaged) LET, rather than as a function of depth. With the exception of the two points at the lowest LET, this revealed a basically linear increase of R_LA_ with LET. Therefore, at least for LET values in the range ~5–25 keV/μm, additional R_LA_ values (where “additional” means in correspondence of additional LET values and, thus, additional depth positions, with respect to those considered in Figures 8 and 9) may be derived by linear interpolation. If the yield of lethal aberrations induced by the same dose of X-rays is known (for instance, from experiments), R_LA_ would then provide the yield of lethal aberrations induced by protons (LA_p_). According to our model, LA_p_ would then allow calculating proton cell survival for these additional depth positions.

**Figure 10:**
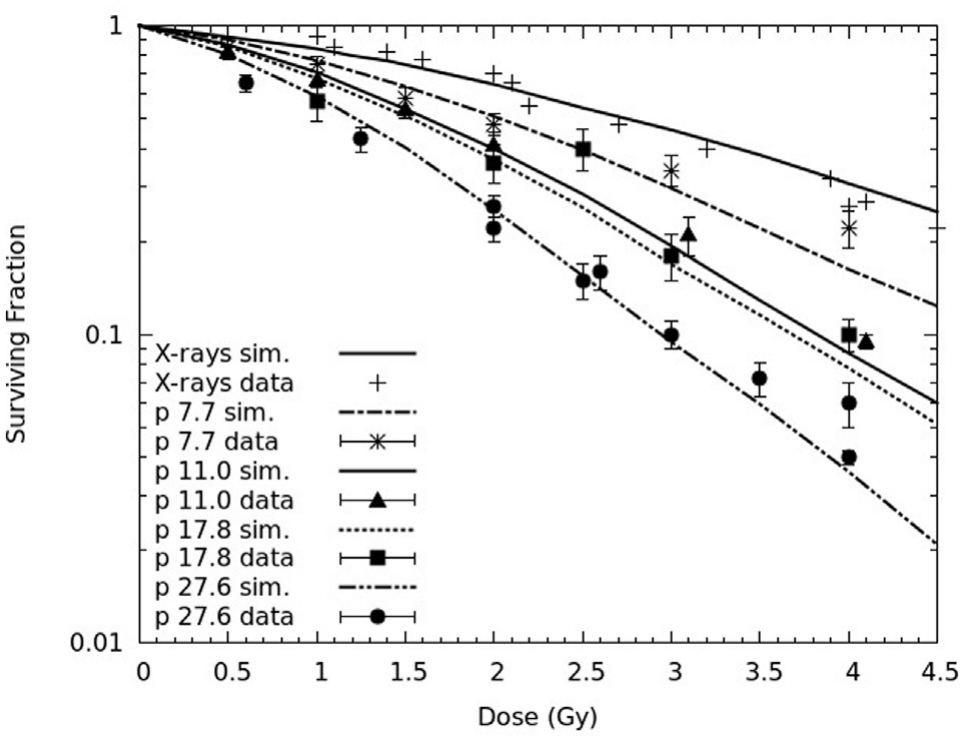
**Survival curves for V79 cells exposed to four monoenergetic proton beams (LET: 7.7, 11.0, 17.8, and 27.6 keV/μm), as well as X-rays as a reference**. The lines are simulation outcomes, the points are experimental data taken from the literature: (32) for X-rays, 7.7 keV/μm protons and 11.0 keV/μm protons; (34) for X-rays, 17.8 keV/μm protons, and 27.6 keV/μm protons.

After considering AG human fibroblasts, the model was applied to V79 hamster fibroblasts, which are rather radio-resistant and are widely used in the characterization of hadron therapy beams. The final goal consisted of predicting cell death and chromosome aberrations for V79 cells along the SOBP dose profile used in Chaudhary et al. (17) to irradiate AG01522 cells. As a preliminary step, to adjust the model parameters before performing such predictions, experimental survival curves taken from the literature for V79 cells exposed to different monoenergetic proton beams, as well as X-rays as a reference (32, 34), were reproduced. Figure 10 reports calculated survival curves for X-rays and four monoenergetic proton beams (with LET values in the range 7.7–27.6 keV/μm), together with experimental data taken from Ref. (32, 34). All the curves reported in Figure 10 were obtained with *f* ≈ 0.1. The difference with respect to the value used for AG cells, which was ~0.2, may be related to the different repair features of these two cell lines. More specifically, AG cells are likely to possess a less efficient repair machinery, implying higher levels of un-rejoined chromosome fragments and, thus, higher *f* values.

Like for AG01522 cells, also for V79 cells the yield of CLs was adjusted separately for each radiation quality, that is for each curve. The X-ray curve reported in Figure 10 was obtained using a CL yield of 1.5 CL Gy^−1^⋅cell^−1^, whereas the four proton curves were obtained with CL yields in the range ~2.0–3.2 CL⋅Gy^−1^⋅cell^−1^, increasing with LET. With these values, the general agreement between the simulation outcomes and the experimental data reported in Ref. (32, 34) was satisfactory. More specifically, for the curve at the lowest LET (7.7 keV/μm), the value of the reduced chi-square was 1.8. Higher (reduced) chi-square values were found for the other curves, for which the maximum relative difference between simulated and measured survival was 47%. However, the maximum relative difference with respect to the data fits provided in (32, 34) was 35%.

Similarly to AG01522 cells, the CL yields used in the present work were lower with respect to previous works in which parameter *f* was not introduced in the model. Furthermore, the CL yields for V79 cells were lower than the CL yields for AG01522 cells exposed to similar radiation qualities, as a consequence of the lower radiosensitivity of V79 cells. In fact, as discussed in detail in previous works [e.g., Ref. (13)], although the CL yield mainly depends on radiation quality, it is also modulated by the specific target cell response. This is consistent with the biophysical meaning of this parameter, which represents a type of DNA damage that is severe and difficult to be repaired.

**Figure 11:**
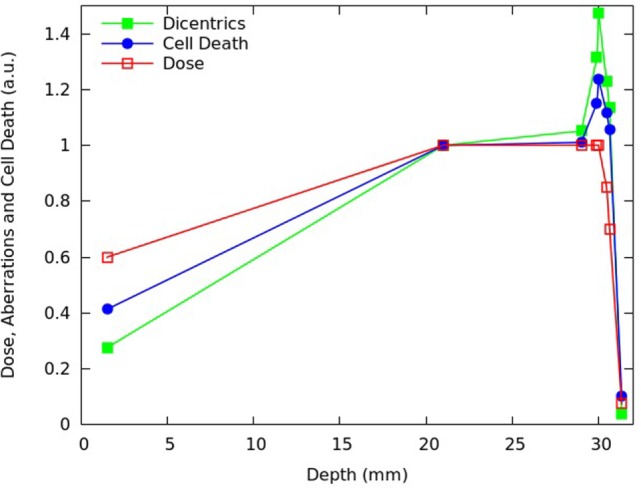
**Predicted fraction of inactivated cells (blue symbols) and mean number of dicentrics per cell (green symbols) for V79 cells at different depths of the SOBP dose profile reported in Chaudhary et al. (17), which is also shown in the figure (red symbols)**. Each quantity was normalized to the proximal point. The lines are simply guides for the eye.

**Figure 12:**
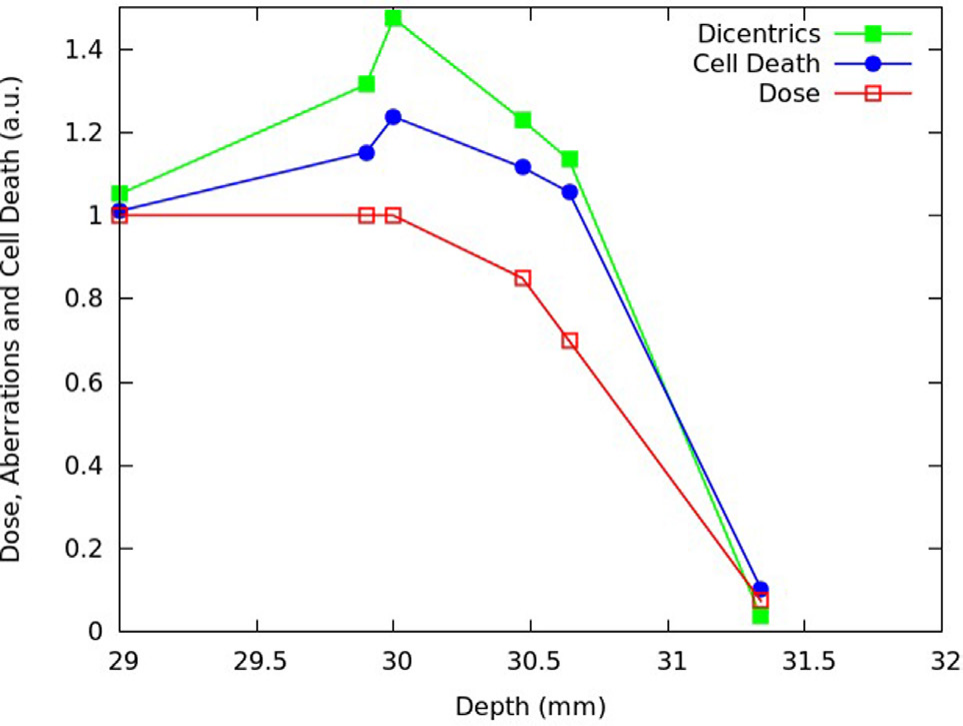
**Distal and fall-off region of the SOBP shown in Figure 11**.

Figures 11 and 12 report predictions of cell death (i.e., fraction of inactivated cells) and chromosome aberrations (i.e., mean number of dicentrics per cell) for V79 cells along the proton SOBP used in Chaudhary et al. (17), as well as the dose profile reported in Chaudhary et al. (17). The results, which were obtained assuming a plateau dose of 2 Gy, were normalized with respect to the proximal position. Among the considered LET values and, thus, the corresponding depth positions, four are those reported in Figure 10, whereas the others [i.e., 3.0, 10.1, and 20.0 keV/μm, for which the survival data for comparison were taken from Ref. (4, 32, 34), respectively] were not reported in Figure 10 to avoid making the figure too crowded.

Like for AG01522 cells, the beam effectiveness was found to increase along the plateau, and high levels of biological damage were also found beyond the distal dose fall-off. Moreover, the increase in chromosome aberrations along the plateau was more pronounced than the increase in cell killing, reflecting the radiation effectiveness dependence on the specific endpoint. Interestingly, the increase in biological effectiveness was more pronounced for V79 cells than for AG01522 cells: for instance, for V79 cells the fraction of inactivated cells increased along the plateau by a factor that was more than 1.2, whereas for AG01522 cells this factor was <1.1. This is consistent with the higher RBE generally shown by cells exhibiting smaller α/β ratios (7), as is the case of V79 cells.

## Conclusion

A biophysical model of radiation-induced cell death and chromosome aberrations, which assumes a pivotal role of DNA cluster damage and lethal aberrations, was further developed and applied to therapeutic protons. After testing an improved version against experimental data, the model was applied to pristine and modulated Bragg peaks of the proton beam used to treat eye melanoma at INFN-LNS in Catania, Italy. Experimental survival curves for AG01522 cells exposed to the Catania beam were reproduced. Cell death and chromosome aberrations were then predicted at different depth positions along a SOBP dose profile, both for AG01522 cells and for V79 cells. In line with other studies, this work indicated that assuming a constant RBE along a proton SOBP may be sub-optimal. Furthermore, the simulations helped quantifying the dependence of the beam effectiveness on the considered endpoint and dose, as well as the cell radiosensitivity.

More generally, this work provides an example of therapeutic beam characterization that is not based on RBE, which can be a source of uncertainties. This approach represents a starting point in view of possible future works in which treatment plan optimization may be directly based on the calculated level of biological effect (typically, fraction of inactivated cells and yields of chromosome aberrations). Of course, to be of practical use, the model should be “coupled” to a TPS and/or a radiation transport code. Moreover, the model should be further refined, e.g., by extending it to other cell lines and correcting the tendency to overestimate the effectiveness at the lower survival levels, if this tendency will be confirmed.

## Author Contributions

MC performed the simulations, interpreted the results, and revised the manuscript. FB designed the work, interpreted the results, and drafted the work. Both authors approved the final version of the manuscript.

## Conflict of Interest Statement

The authors declare that the research was conducted in the absence of any commercial or financial relationships that could be construed as a potential conflict of interest.

## Footnotes

^1^http://www.ptcog.ch/
